# Validity and Reliability of Inertial Measurement Unit (IMU)-Derived 3D Joint Kinematics in Persons Wearing Transtibial Prosthesis

**DOI:** 10.3390/s23031738

**Published:** 2023-02-03

**Authors:** Jutima Rattanakoch, Manunchaya Samala, Weerawat Limroongreungrat, Gary Guerra, Kittichai Tharawadeepimuk, Ampika Nanbancha, Wisavaporn Niamsang, Pichitpol Kerdsomnuek, Sarit Suwanmana

**Affiliations:** 1 Sirindhorn School of Prosthetics and Orthotics, Faculty of Medicine Siriraj Hospital, Mahidol University, Bangkok 10700, Thailand; 2 College of Sports Science and Technology, Mahidol University, Nakhon Pathom 73170, Thailand; 3 Exercise and Sport Science Department, St. Mary’s University, San Antonio, TX 78228, USA; 4 Department of Orthopaedic Surgery, Faculty of Medicine Siriraj Hospital, Mahidol University, Bangkok 10700, Thailand

**Keywords:** inertial measurement unit (IMU), optical motion capture (OMC), intra- and inter-subject variability, transtibial prosthesis, gait parameters assessment

## Abstract

Background: A validity and reliability assessment of inertial measurement unit (IMU)-derived joint angular kinematics during walking is a necessary step for motion analysis in the lower extremity prosthesis user population. This study aimed to assess the accuracy and reliability of an inertial measurement unit (IMU) system compared to an optical motion capture (OMC) system in transtibial prosthesis (TTP) users. Methods: Thirty TTP users were recruited and underwent simultaneous motion capture from IMU and OMC systems during walking. Reliability and validity were assessed using intra- and inter-subject variability with standard deviation (S.D.), average S.D., and intraclass correlation coefficient (ICC). Results: The intra-subject S.D. for all rotations of the lower limb joints were less than 1° for both systems. The IMU system had a lower mean S.D. (^o^), as seen in inter-subject variability. The ICC revealed good to excellent agreement between the two systems for all sagittal kinematic parameters. Conclusion: All joint angular kinematic comparisons supported the IMU system’s results as comparable to OMC. The IMU was capable of precise sagittal plane motion data and demonstrated validity and reliability to OMC. These findings evidence that when compared to OMC, an IMU system may serve well in evaluating the gait of lower limb prosthesis users.

## 1. Introduction

Amputation has a marked effect on an individual’s quality of life (QOL) [1], yet it is well understood that a prosthesis can facilitate a return to a higher quality of life [2]. Returning to an ambulant lifestyle and activities of daily living is the cornerstone of prosthetic intervention [3]. Mobility is the most important factor related to QOL in lower limb prosthesis users [4]. It is the responsibility of the prosthetist and rehabilitation team to make informed and customized choices for a suitable prosthetic prescription based on patient preference and practitioner expertise. Provision of an acceptable continuum of care requires pre- and post-prosthetic outcome measurement and continuous long-term ambulatory measurements. Evaluating the effects of a prosthetic intervention on a prosthesis user’s quality of life is conducted using biomechanical outcome measurements. Adequately measuring the outcome of prosthetic interventions is an important and recommended procedure for ensuring a continuum of quality routine clinical care [5].

An inertial measurement unit (IMU) is a gyroscope, accelerometer, and magnetometer combined into a small wearable unit [6]. These wearables can be placed on specific anatomical locations of the body and integrated with software to calculate real-time free-living joint kinematics [7,8,9]. 

IMU technology is portable because it does not require a set camera infrastructure and is simple to apply in many situations [10]. As such, this technology is ideal for human biomechanics research due to its portability, low cost, and low power consumption [11]. Using an IMU affords wireless joint angle determination and Bluetooth transmission to a computer for further filtering and analysis [12]. These IMU monitors have been evaluated for accuracy against gold-standard motion capture analysis with positive results [13]. A systematic review concluded that inertial sensors could provide an accurate and reliable method for studying human motion, although the degree of accuracy and reliability varies by sensor location and task [11].

There are several commercially-available wearables, but only some devices have been deemed accurate and reliable for non-disabled persons [14,15,16]. Even fewer devices have been tested for the accuracy of walking in the lower extremity amputee (LEA) population, and there are currently very few joint angular kinematic studies [17,18,19,20]. Few studies have utilized IMU to evaluate lower limb prosthetics to date. Previous studies have examined pelvis or trunk parameters which may assist the analysis of gait balance and symmetry [21]. The utility of wearables is their potential to measure activity related to specific prosthetic interventions [22]. Due to the portability of IMUs, important metrics about the free-living activity can be derived [14]. The primary objective of this study was to evaluate the accuracy and reliability of IMUs on walking kinematics of transtibial prosthesis (TTP) users against a criterion optical motion capture (OMC) system.

## 2. Materials and Methods

### 2.1. Participants

**Figure 1:**
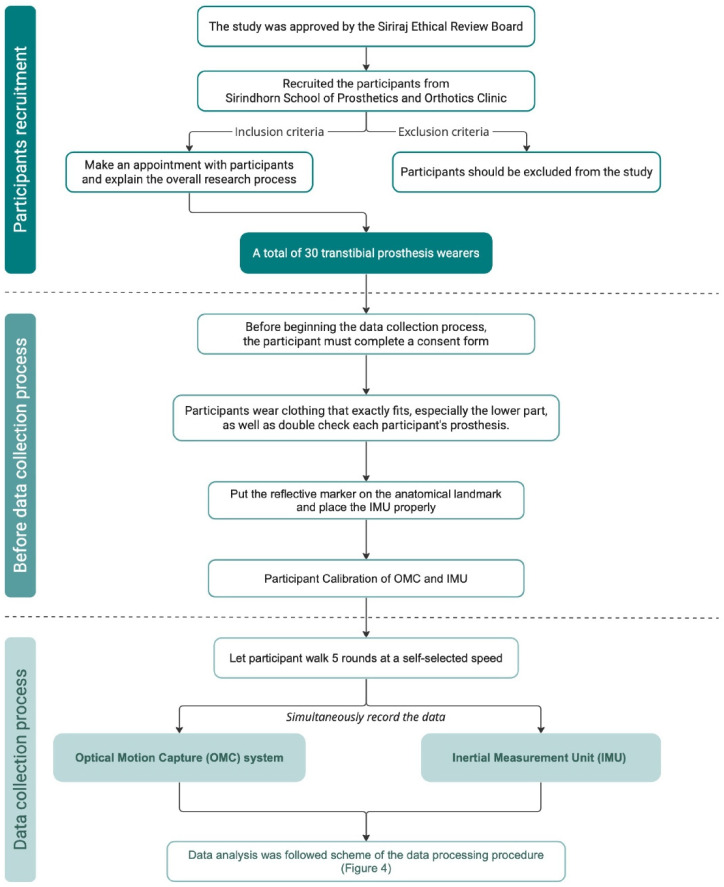
Flow chart showing the experimental design of the study.

All participants were recruited from the Sirindhorn School of Prosthetics and Orthotics Clinic. The Siriraj Ethical Review Board approved study number 892/2564 (IRB3)**,** and all participants provided consent prior to any data collection. A total of 30 transtibial prosthesis wearers (25 males and 5 females), ages 53 ± 12 years, body weight 73.12 ± 26.7 kg, and height 1.59 ± 24.8 m, participated in this study. The sample size determination followed methods described in previous literature exploring sensor accuracy [13,23]. All participants were in good health, could walk without a cane or other aid, and had a normal range of motion in their lower limb joints. The prosthetist checked each prosthesis to ensure it was functioning optimally and that it was safe for the person to perform the study procedures. A methodological flowchart is shown in Figure 1 to illustrate the experimental design of the study. 

### 2.2. Instrumentation and Protocol

**Figure 2:**
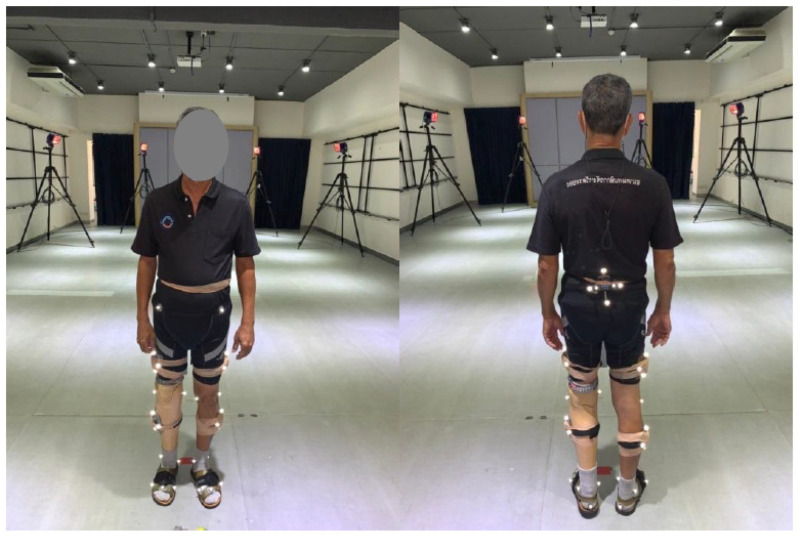
Image displaying a single comprehensive set for two systems, Inertial measurement unit (IMU) and cluster-based marker (CBM) set of optical motion capture system (OMC).

**Figure 3:**
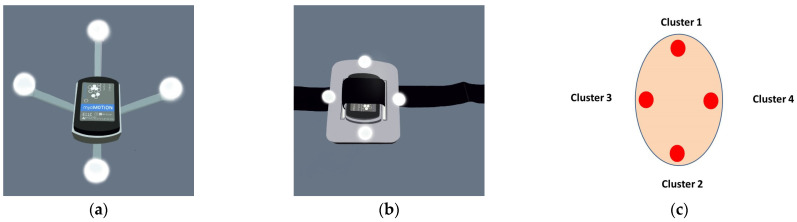
Images (**a**,**b**) are the rigid plates for IMU and CBM set of OMC (**c**). The cluster-based marker (CBM) set for an optical motion capture (OMC). Both the IMU and the cluster were built with the same coordinate system.

Simultaneous data capture was accomplished by combining both systems, as shown in Figure 2 and Figure 3. To attach the IMU and cluster-based marker (CBM) for OMC, a 3D-printed marker plate combining markers and the IMU was developed (Figure 3). This rigid plate permitted optimal marker and IMU alignment. The cluster-based marker (CBM) and strap-on IMUs were placed on each subject (Figure 2). The IMU system was the Noraxon MyoMotion Research Pro system (Noraxon USA, Scottsdale, AZ) with a sampling rate of 200 Hz. Seven IMUs were attached using Velcro straps at the feet, legs, thighs, and pelvis. The IMU MyoRESEARCH 3.8.1 software was used to collect data. The five-cluster and 40-marker six degree of freedom retro-reflective marker set (20.1 mm) were used [24,25,26]. Eight Raptor series cameras recorded markers (Motion Analysis Corporation, Santa Rosa, CA, USA) sampling at 200 Hz. The five rigid plates combined with the CBM permitted optimal marker and IMU alignment. Additional markers were placed at ASIS, PSIS, the medial and lateral knee joint centers, the medial and lateral malleoli, the 1st, 2nd, and 5th heads of the metatarsals, and the center of the heel. Markers were placed at anatomically relevant locations on the prosthetic side. A double-sided tape was also used for the IMU and markers to minimize movement. All IMU with cluster-based marker (CBM) plates and retro-reflective markers were positioned by a single rater who was a professional prosthetist with extensive motion analysis training. The recording of both IMU data and motion capture marker data started and stopped via a synchronized timing pulse so that all measurements of IMU data and mocap marker data coincided.

A static standing trial was performed to gather a baseline for dynamic trials with the IMU and OMC systems. Before the dynamic walking trial, the IMU system was calibrated while standing. Participants performed five repeated walking trails in a single direction along a flat indoor walkway (10 m), with 300 left and right gait cycles used for analysis. Before each data collection, the IMU system was recalibrated to ensure no sensor drift. A synchronized timing pulse was used to start and end the recording of the IMU data as well as OMC data, ensuring that all measurements of the two types of data were synced and accurate.

Foot strike events were obtained for OMC using the lowest position of heel detection [27], and for IMU using foot acceleration signals [28]. Moreover, high-speed cameras and gait timers were used to visually confirm the gait initiation and even detection of the OMC and IMU systems. Through the alignment of kinematic events, IMU data and OMC data were brought into sync with one another with respect to time. 

### 2.3. Data Processing

For IMU and OMC, hip, knee, and ankle gait angles were calculated separately for each data set using the gait analysis software package for those systems.

Data processing for OMC trials was performed with Cortex software (Motion Analysis Corporation, Santa Rosa, CA, USA). A fourth-order, zero-lag, low-pass Butterworth filter (6 Hz) was used to smooth trajectory paths. Marker data were then modeled in Visual 3D (C-Motion Inc., Germany, MD, USA) and utilized for joint angle calculations using a Cardan sequence of rotations [29].

Data processing for IMU trials was performed with the MyoRESEARCH 3.8.1 software. The position and velocity of the IMUs in each phase of movement were calculated using the MyoRESEARCH 3.8.1 software (Noraxon USA, Scottsdale, AZ). The lower limb joint angle can be calculated according to the Cardan rotation sequence, following the International Society of Biomechanics (ISB) recommendations [30]. 

Joint angles measured using IMU integration typically drift over time [31]. To overcome the drift problem that occurs in an IMU sensor, the use of accelerometers and magnetometers in a complimentary filter and tracking algorithms that limit limb movement is frequently employed [12]. The MyoRESEARCH 3.8.1 software has incorporated robust stabilization and the appropriate algorithms to prevent potential sensor issues [32].

**Figure 4:**
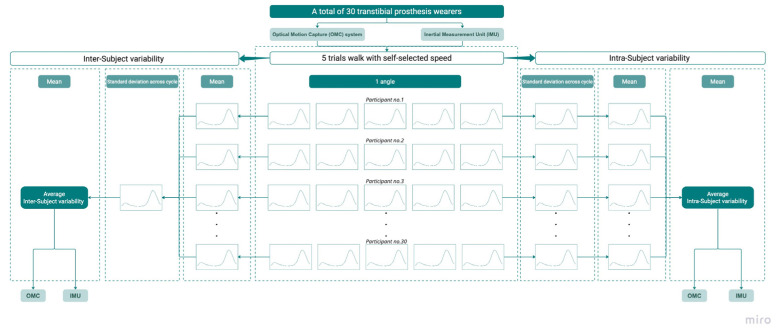
Procedure of data processing [26] for averaged intra-subject variability and average inter-subject variability across the gait cycle.

To determine intra-subject variability, the standard deviation (S.D.) was calculated from five gait trial repetitions for each percentage point of the gait cycle and averaged over the cycle to give the mean and S.D. within each subject for that specific kinematic variable using the procedure illustrated in Figure 4. The mean S.D. of each participant during the gait cycle was used to obtain the average intra-subject variability across 30 subjects.

The average joint rotation of each participant was computed from five gait trial repetitions over each percentage point of the gait cycle and used to compute the within-group S.D. for 30 participants. This was then averaged across the gait cycle to calculate the inter-subject variability as illustrated in Figure 4. 

## 3. Results

### 3.1. IMU and OMC System Comparison (Intra-Subject Variability and Inter-Subject Variability)

The repeatability of joint angle calculations for each subject was summarized in the value of the standard deviation of each joint angle during the gait cycle to allow an overall investigation of intra-subject variability and inter-subject variability. These average values are reported in Table 1 and Table 2.

In Table 1, the intra-subject variability, as reported by the average S.D. (in degrees), was below 5.00 for all rotations of the lower limb joint on the amputated side in subjects for both systems. 

In particular, the intra-subject variability of hip rotation on the prosthetic side was an average of 0.18° for IMU and 0.14° for OMC. The average values represented the intra-subject variability of knee rotation for IMU and OMC, 0.34° and 0.17°, respectively. For the ankle joint, the intra-subject variability was an average of 0.34° for IMU and 0.08° for OMC.

Similar information is included in Table 2 for the sound lower limb joint; the maximum average value was 0.55° for all rotations of the lower limb joint on the sound side in subjects for both systems. In particular, the intra-subject variability of hip rotation on the sound side was an average of 0.13° for IMU and 0.13° for OMC. The knee rotation of all participants had an intra-subject variability with average values for IMU and OMC of 0.26° and 0.14°, respectively. The intra-subject variability for all subjects for the ankle joint was an average of 0.29° for IMU and 0.16° for OMC.

For both techniques of measuring kinematics of the hip and knee joints on both sides, there was good intra-subject repeatability among the measured variables. On the amputated side, the average difference in degrees between the two systems for the ankle joint was greater than 2 degrees, whereas on the sound side, the difference was fairly low. Overall, both systems showed good within-subject repeatability across all degrees of freedom for all joints.

As indicated in Table 1 and Table 2, inter-subject variability was determined using the mean degree (°) of the standard angle deviation over the amputation and sound gait cycles among the thirty individuals for both systems. On the amputated side, the between-subject variability for IMU varied from 1.93° to 7.50°, with an average of 4.77°, and for OMC, it ranged from 2.54° to 8.17°, with an average of 4.77°. On the sound side, IMU ranged from 1.99° to 6.43° with an average of 4.67°, whereas the OMC ranged from 3.26° to 8.22° with an average of 5.26°. The mean standard deviation for the OMC and IMU was similar on the amputated side but was greater for the OMC than the IMU on the sound side. The overall difference between subjects for the IMU was about 4.70° for both the amputated limb and the sound limb. 

### 3.2. Intraclass Correlation Coefficients (ICCs)

The intraclass correlation coefficient (ICC) is a frequently used measure of reliability in interrater research. An ICC assessment of r > 0.75 is considered to have good reliability, r = 0.40–0.75 fair-to-good reliability, and r = 0.40 poor reliability [33]. As shown in Table 3, the agreement between the IMU and OMC models was good-to-excellent for all kinematic parameters in the sagittal plane, with a range of 0.60 to 0.99. The knee flexion/extension of both limbs had the highest reliability (>0.9), followed by hip flexion/extension, ankle dorsiflexion, and plantarflexion. For the frontal plane, ankle inversion/eversion on both sides and knee abduction/adduction on the amputated side demonstrated poor reliability. On the sound side, however, knee abduction/adduction reliability was fair, while hip abduction/adduction reliability on both sides was excellent. In the transverse plane, poor reliability of the kinematics of three joints was observed, as indicated by r values less than 0.40.

### 3.3. Key Kinematic Parameter

A paired t-test with a significance level of 0.05 was used to compare key kinematic parameters over the gait cycle for IMU and OMC systems, which are represented in Table 4, Table 5 and Table 6. There were significant differences in maximum hip flexion and extension on both the amputated and sound side, except for hip abduction and adduction, which were similar. On the other hand, the average hip range of motion in the sagittal plane was not different on the sound side. There were no significant differences in the sagittal plane for knee kinematics on the amputated side; however, there were significant differences on the sound side, with exception of maximum knee flexion. Interestingly, ankle motions were similar in the sagittal and frontal planes on both amputated and sound sides.

### 3.4. Agreement between IMU and OMC Systems by Virtual Waveform Comparison

**Figure 5:**
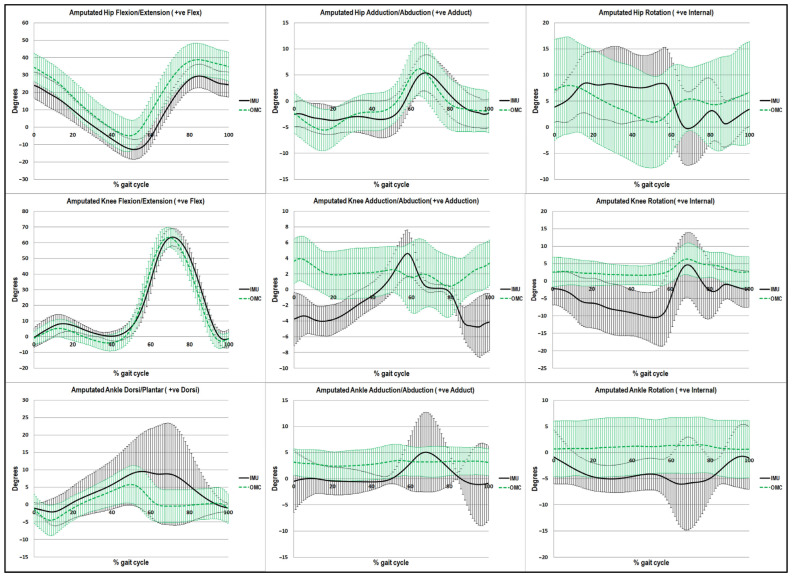
The visual waveform of walking kinematics on the amputated side. The black lines represent IMU and the green lines represent OMC. Note: IMU: Inertial Measurement Unit, OMC: Optical Motion Capture.

**Figure 6:**
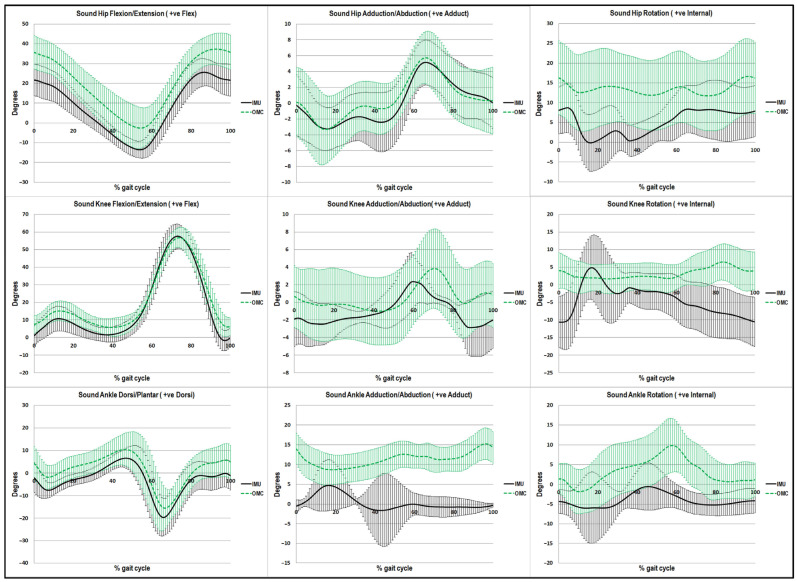
The visual waveform of walking kinematics on the sound side. The black lines represent IMU and the green lines represent OMC. Note: IMU: Inertial Measurement Unit, OMC: Optical Motion Capture.

Figure 5 and Figure 6 illustrate nine kinematic variables which were analyzed for the two systems by plotting the mean gait cycle plus or minus one standard deviation. On the amputated and sound sides in Figure 5 and Figure 6, respectively, the kinematic waveforms were visually examined in the sagittal, coronal, and transverse planes. The figures provide visual comparisons of kinematic data for the entirety of the gait cycle between systems.

In each plane, Figure 5 and Figure 6 illustrate the IMU and OMC joint angles for the hip, knee, and ankle. The shaded black area represents a mean ± 1 S.D. from IMU, while the shaded green area represents a mean ± 1 S.D. from OMC. Particularly for the joint motion at the hip and knee on both sides as well as the ankle motion on the sound side, the graphs in the sagittal plane provide good illustrations of agreement between the two systems. There was a wide range of ankle motion on the amputated side depending on the type of prosthetic foot.

## 4. Discussion

The purpose of this study was to examine the walking joint kinematics of two different motion analysis systems in the gait of 30 transtibial prosthesis wearers. 

Intra-subject variability for kinematic results was observed and ranged from 0.04 to 0.72 across the two systems on the amputated side. Although slightly higher average standard deviation values were observed in the IMUs, this was considered reasonable and did not exceed 1° [34,35]. The average standard deviations of the hip, knee, and ankle on three planes were 0.13°, 0.26°, and 0.29°, respectively, while they were 0.13°, 0.14°, and 0.16° for the OMC system. Both systems showed strong within-subject repeatability for all joints and degrees of freedom, particularly when compared to the standard deviation seen previously observed in the OMC system, which ranged from 0.90° to 4.60° [36].

The inter-subject variability represented the mean of the standard deviation, and it was greater for the OMC system than the IMU system for the majority of parameters, with the exception of knee internal/external rotation, ankle dorsi/plantarflexion, and ankle adduction/abduction on the amputated side. Similar to the sound side, all parameters in the OMC system except for knee flexion/extension, knee internal/external rotation, and ankle adduction/abduction showed higher standard deviations than those in the IMU. This demonstrated that there was a smaller mean (°) of standard deviation among the thirty participants in the IMU system, particularly for hip and knee joint motions. Additionally, for both the sound limb and the amputated limb, the average difference between subjects for the IMU was less than 5° [34]. The IMU system had a smaller mean (°) standard deviation than the OMC system according to this study and a prior study [36].

Moreover, a review of prior studies comparing the validity, reliability, and precision of inertial sensors to OMC observed errors between 2 and 5 degrees, which are regarded as acceptable [37].

According to the intraclass correlation coefficient, the agreement between the IMU and OMC models was good to excellent for all kinematic parameters in the sagittal plane. The highest reliability was found in limb knee flexion and extension, followed by hip flexion and extension, ankle dorsiflexion, and plantarflexion. This is observed in kinematic graphs, which provide a means to compare and interpret quantitative 3D gait data [38]. Visually comparing both kinematic waveforms showed the IMU system had good waveform agreement, especially in the sagittal plane. The frontal and transverse plane kinematic parameters on the amputated and sound gait cycles had the lowest consistency of general patterns for ankle rotation, which may be a result of reflecting the variation in participant prosthetic foot and movement function on the amputated side. Regarding the main kinematic parameters during the gait cycle, there were no significant differences in the sagittal plane for the knee kinematics on the amputated side. On the sound side, however, there were significant differences, except for maximum knee flexion. Kinematics of the ankle in the sagittal and frontal planes were similar on both sides.

## 5. Conclusions

From all kinematic comparisons between the IMU and OMC systems, we can surmise that the IMU’s kinematic output is comparable to that of the OMC. The IMU can provide accurate sagittal plane motion data for transtibial prosthetic gait analysis. Moreover, the IMU can more easily assess gait than the OMC, with a reduced setup time and cost of equipment. However, outside of the sagittal plane, the ICC has less agreement. Nonetheless, the findings of this study demonstrated that, when compared to the standard OMC system, the IMU can provide an accurate result and is a practical system for measuring amputee gait. This result has important implications for sensor-based motion analysis for clinical populations in need of objective biomechanical analysis. IMUs for motion analysis research are emerging as effective gait analysis tools outside the confines of the motion analysis laboratory for transtibial prosthesis users. Further research should concentrate on evaluating amputee gait using IMU with kinematic analysis for walking on uneven terrain, ascending or descending stairs, playing sports, or participating in other activities outside of the laboratory. These technologies may offer a means for the evaluation of prosthesis rehabilitation in both laboratory and free-living settings.

## Figures and Tables

**Table 1:** Average intra-Subject variability and inter-subject variability over the amputee gait cycle among the thirty participants for both systems.

Rotation (°)	Intra-Subject Variability	Intra-Subject Variability	Inter-Subject Variability	Inter-Subject Variability
	IMU	OMC	IMU	OMC
Hip Flex/Extension	0.29	0.32	5.76	8.17
Hip Ab/Adduction	0.08	0.05	2.91	3.73
Hip Int/External	0.17	0.12	5.42	7.73
Knee Flex/Extension	0.72	0.41	4.74	5.61
Knee Ab/Adduction	0.14	0.05	1.93	2.98
Knee Int/External	0.17	0.06	6.54	3.33
Ankle Flex/Extension	0.37	0.13	7.49	4.03
Ankle Ab/Adduction	0.32	0.04	3.95	2.54
Ankle Int/External	0.32	0.07	4.14	4.85

**Table 2:** Average intra-subject variability and inter-subject variability over the sound gait cycle among the thirty participants for both systems.

Rotation (°)	Intra-Subject Variability	Intra-Subject Variability	Inter-Subject Variability	Inter-Subject Variability
	IMU	OMC	IMU	OMC
Hip Flex/Extension	0.14	0.17	5.87	7.83
Hip Ab/Adduction	0.09	0.08	2.85	3.28
Hip Int/External	0.15	0.15	5.46	8.22
Knee Flex/Extension	0.55	0.32	5.80	5.31
Knee Ab/Adduction	0.08	0.04	1.99	3.58
Knee Int/External	0.14	0.07	6.43	4.01
Ankle Flex/Extension	0.30	0.18	5.57	6.65
Ankle Ab/Adduction	0.32	0.10	3.95	3.26
Ankle Int/External	0.25	0.21	4.09	5.21

**Table 3:** Between-protocol intraclass correlation coefficients (ICC) for lower limb measures during the gait cycle.

Parameters	Amputated Side	Sound Side
Hip Flexion/Extension	0.90	0.84
Hip Abduction/Adduction	0.94	0.97
Hip Rotation	−0.62	0.05
Knee Flexion/Extension	0.99	0.98
Knee Abduction/Adduction	−0.16	0.52
Knee Rotation	0.24	−0.28
Ankle Dorsiflexion/Plantarflexion	0.60	0.88
Ankle Inversion/Eversion	0.11	−0.07
Ankle Rotation	0.05	0.21

**Table 4:** Hip joint angle parameters of thirty participants on sound and amputated side as mean (S.D.) over five gait cycles calculated by the IMU and OMC.

Parameters	IMU (S.D.)	OMC (S.D.)	*p*-Value	*p* < 0.05	*p* < 0.001
**Sound side**					
Hip Flex/Extension ROM	38.40 (3.10)	38.90 (4.70)	0.24		
Peak Stance Extension	−15.00 (4.20)	2.10 (9.30)	0.00	*	**
Peak Swing Flexion	23.40 (6.00)	41.00 (7.70)	0.00	*	**
Hip Ab/Ad ROM	9.80 (1.70)	10.40 (3.30)	0.26		
Hip Int/Ext Rotation ROM	13.90 (4.70)	8.00 (2.90)	0.00	*	**
**Amputated side**					
Hip Flex/Extension ROM	42.70 (6.50)	43.70 (6.00)	0.00	*	**
Peak Stance Extension	−14.80 (5.80)	−0.60 (10.10)	0.00	*	**
Peak Swing Flexion	28.00 (6.60)	43.00 (9.50)	0.00	*	**
Hip Ab/Ad ROM	10.80 (3.00)	12.00 (2.80)	0.29		
Hip Int/Ext Rotation ROM	13.20 (4.70)	9.40 (3.80)	0.00	*	**

* Significant difference (α = 0.05), ** Indicates significance level of *p* < 0.001 after Bonferroni correction (0.05/30).

**Table 5:** Knee joint angle parameters of thirty participants on sound and amputated side as mean (S.D.) over five gait cycles calculated by the IMU and OMC.

Parameters	IMU (S.D.)	OMC (S.D.)	*p*-Value	*p* < 0.05	*p* < 0.001
**Sound side**					
Knee Flex/Extension ROM	58.10 (5.00)	52.10 (6.30)	0.00	*	**
Peak Stance Extension	−2.70 (5.30)	4.40 (4.90)	0.00	*	**
Peak Swing Flexion	55.50 (6.20)	56.60 (5.50)	0.29		
Knee Ab/Ad ROM	7.40 (4.40)	5.10 (2.20)	0.01	*	
Knee Int/Ext Rotation ROM	19.10 (5.60)	6.70 (2.30)	0.00	*	**
**Amputated side**					
Knee Flex/Extension ROM	67.80 (4.80)	69.2 (10.7)	0.62		
Peak Stance Extension	−4.70 (3.60)	−5.70 (7.00)	0.57		
Peak Swing Flexion	63.20 (4.70)	63.50 (8.40)	0.83		
Knee Ab/Ad ROM	11.70 (6.10)	6.60 (2.50)	0.00	*	**
Knee Int/Ext Rotation ROM	19.40 (5.70)	5.60 (3.50)	0.00	*	**

* Significant difference (α = 0.05), ** Indicates significance level of *p* < 0.001 after Bonferroni correction (0.05/30).

**Table 6:** Ankle joint angle parameters of thirty participants on sound and amputated side as mean (S.D.) over five gait cycles calculated by the IMU and OMC.

Parameters	IMU (S.D.)	OMC (S.D.)	*p*-Value	*p* < 0.05	*p* < 0.001
**Sound side**					
Ankle Plantar/Dorsiflexion	28.10 (7.80)	29.90 (6.00)	0.66		
Peak Stance Dorsiflexion	10.70 (7.80)	12.70 (6.10)	0.20		
Peak Swing Plantarflexion	−20.50 (10.60)	−17.50 (8.50)	0.07		
Ankle Ab/Adduction	11.00 (17.10)	8.70 (2.70)	0.06		
Ankle Inv/Eversion ROM	16.00 (6.40)	14.00 (5.10)	0.05	*	
**Amputated side**					
Ankle Plantar/Dorsiflexion	17.00 (14.00)	11.70 (6.70)	0.19		
Peak Stance Dorsiflexion	12.00 (16.20)	8.30 (1.70)	0.25		
Peak Swing Plantarflexion	−2.70 (3.10)	−3.90 (7.00)	0.28		
Ankle Ab/Adduction	10.90 (16.80)	2.50 (2.30)	0.03	*	
Ankle Inv/Eversion ROM	16.00 (6.50)	1.70 (2.20)	0.00	*	**

* Significant difference (α = 0.05), ** Indicates significance level of *p* < 0.001 after Bonferroni correction (0.05/30).
